# Review of "HIV Chemotherapy: a Critical Review" by Salvatore T. Butera

**DOI:** 10.1186/1742-6405-3-7

**Published:** 2006-03-23

**Authors:** J Scott Cairns

**Affiliations:** 1 Henry M Jackson Foundation

Don't let the title fool you, this book gives a wide view of the state of the field in HIV treatment world-wide and includes some enticing discussions of potential targets and strategies on the horizon. Given its wide range of topics and its focus on more than just small molecule inhibitors of HIV, the title seems almost too restrictive. The book is divided into 4 areas: Issues in Clinical Management, Issues Related to Drug Resistance, Implementation in Developing Countries, and New Antiviral Targets. As such, it could be a good source of information for investigators new to the field or those needing a quick source of information in areas tangential to their main focus. As is the case with many textbooks that attempt to describe rapidly moving fields, many of the areas covered in the book contain newer information, and readers are advised to investigate more recent publications in areas they may find of interest.

With its emphasis on drug resistance, toxicity, and complex dosing strategies, the section on clinical management sets the stage for the remainder of the book by making the case for the need for additional drugs and other anti-viral strategies. A chapter on immune-based therapies was also included in this section, focusing on clinical trial results that involved the use of various cytokines to affect immune and viral parameters. More attention here and in the last section could have been paid to different delivery systems as well as to strategies that target the innate immune system. The third chapter in this section, on the use of treatment interruption strategies to manage HIV infection gave a complicated picture of the relevance of this strategy in the HIV treatment armamentarium. More recent studies of this approach, such as the recently released interim results of the SMART study, only serve to confirm that risks associated with treatment interruption need to be considered in the design of any trial that incorporates this strategy.

The section on drug resistance was comprehensive to this unpracticed eye. The extensive discussions on resistance mutations in chapter 4 were reiterated to a large extent in the following chapter. Here editorial assistance would have been of value in paring down this information and omitting redundancies. I found the section in chapter 4 on lethal mutagenesis as a potential therapeutic strategy to be particularly interesting and recent additions to the literature point to KP-1212 as a mutagenic nucleoside with favorable activity in vitro. Conflicting accounts of possible mitochondrial toxicity point to the need for closer examination of this issue.

Chapters 6 and 7 gave informative overviews of the difficulties inherent in implementing HIV treatment in developing countries. The chapter on prevention of mother-to-child transmission was particularly enlightening.

The final section on new antiviral targets includes a comprehensive overview of entry inhibitors, and a discussion of inhibitors of host factors. I found the latter discussion to have been confusingly written in passages, missing in certain critical details (what were the effects of NAC on viral load, a critical parameter in the testing of any drug with potential anti-viral activities) and in need of editorial assistance. But it left the reader with the correct impression of the relative lack of good, non-toxic inhibitors of host factors. This section omits reference to recently released data on promising phase I studies on the maturation inhibitor PA-457, the first of its class to enter clinical trials. The chapter on RNAi is a nice summary of the state of the art in this rapidly emerging field. As was pointed out by the authors, given the specificity of these molecules and the inherent difficulties in delivering them to the cells of interest, it is unclear whether this strategy will have an impact as a therapy on a virus as heterogeneous and rapidly changing as HIV. What was quite clear was the value of this approach as a basic research tool to examine the role of the factor of interest. The final chapter, on clearance of the latent reservoir, pointed out the difficulties inherent in deleting this long-lived reservoir of infected cells. Given that these cells do not transcribe or express viral proteins in a way that is accessible to current retroviral inhibitors or immune-based strategies, the challenges are indeed great but must be met if HIV is ever to be eradicated from the infected individual. Promising but preliminary results of a recent clinical trial of the DNA remodeling drug valproic acid were not discussed.

In sum, this book gives a balanced account of issues related to the clinical management of HIV in both developed and developing countries. Additional glimpses are provided of new developments of possible relevance to HIV treatment. These offer hope that the pipeline of new drugs and therapies will continue to expand to meet the growing need imposed by the development of resistance and toxicities associated with current therapies.

